# SNPs in Mammary Gland Epithelial Cells Unraveling Potential Difference in Milk Production Between Jersey and Kashmiri Cattle Using RNA Sequencing

**DOI:** 10.3389/fgene.2021.666015

**Published:** 2021-08-03

**Authors:** Syed Mudasir Ahmad, Basharat Bhat, Shakil Ahmad Bhat, Mifftha Yaseen, Shabir Mir, Mustafa Raza, Mir Asif Iquebal, Riaz Ahmad Shah, Nazir Ahmad Ganai

**Affiliations:** ^1^Division of Animal Biotechnology, Faculty of Veterinary Sciences and Animal Husbandry, Sher-e-Kashmir University of Agricultural Sciences and Technology, Srinagar, India; ^2^Division of Food Science, Faculty of Horticulture, Sher-e-Kashmir University of Agricultural Sciences and Technology, Srinagar, India; ^3^Division of Animal Genetics and Breeding, Faculty of Veterinary Sciences and Animal Husbandry, Sher-e-Kashmir University of Agricultural Sciences and Technology, Srinagar, India; ^4^Centre for Agricultural Bioinformatics (CABin), ICAR-Indian Agricultural Statistics Research Institute, New Delhi, India; ^5^Directorate Planning and Monitoring, Sher-e-Kashmir University of Agricultural Sciences and Technology, Srinagar, India

**Keywords:** Jersey and Kashmiri cattle, SNP identification, mammary epithelial cells, RNA Seq data, milk production

## Abstract

Deep RNA sequencing experiment was employed to detect putative single nucleotide polymorphisms (SNP) in mammary epithelial cells between two diverse cattle breeds (Jersey and Kashmiri) to understand the variations in the coding regions that reflect differences in milk production traits. The low milk-producing Kashmiri cattle are being replaced by crossbreeding practices with Jersey cattle with the aim of improving milk production. However, crossbred animals are prone to infections and various other diseases resulting in unsustainable milk production. In this study, we tend to identify high-impact SNPs from Jersey and Kashmiri cows (utilizing RNA-Seq data) to delineate key pathways mediating milk production traits in both breeds. A total of 607 (442 SNPs and 169 INDELs) and 684 (464 SNPs and 220 INDELs) high-impact variants were found specific to Jersey and Kashmir cattle, respectively. Based on our results, we conclude that in Jersey cattle, genes with high-impact SNPs were enriched in nucleotide excision repair pathway, ABC transporter, and metabolic pathways like glycerolipid metabolism, pyrimidine metabolism, and amino acid synthesis (glycine, serine, and threonine). Whereas, in Kashmiri cattle, the most enriched pathways include endocytosis pathway, innate immunity pathway, antigen processing pathway, insulin resistance pathway, and signaling pathways like TGF beta and AMPK which could be a possible defense mechanism against mammary gland infections. A varied set of SNPs in both breeds, suggests a clear differentiation at the genomic level; further analysis of high-impact SNPs are required to delineate their effect on these pathways.

## Introduction

Cow milk is an essential natural product which provides a medium for nutrients including growth and immune factors to offspring and a valued raw material for human food (Séverin and Wenshui, 2005; Reinhardt and Lippolis, 2006). It plays an important role in supporting a healthy immune system and provides protection against infections (Goldman, 2000; Séverin and Wenshui, 2005). Milk is produced in the gland by mammary epithelial cells (MEC), which are gradually exfoliated from the epithelium during lactation (Boutinaud et al., 2015). Bovine milk comprises a diverse population of somatic cells including epithelial cells, macrophages, neutrophils, and lymphocytes. The process of lactation in animals consists of several physiological and metabolic changes (Arora et al., 2019). The length of lactation and yield greatly varied among the breeds (Auldist et al., 2007; Mech et al., 2008). Augmenting milk production in cattle is an essential step toward improving the profitability of dairy farms, and the success of dairy forms plays a crucial role in ensuring economic sustainability.

Single nucleotide polymorphisms markers are being rapidly used in selective breeding programmers for improving phenotypic selections of the animals through genomic selection (GS), gene-assisted selection (GAS), and marker-assisted selection (MAS) methods (Georges et al., 1995; Dekkers, 2004; Hayes and Goddard, 2010). It is remarkable that the discovery of the SNP for economic traits has great potential in the genetic improvement of cattle (Pareek et al., 2017). One of the great interests is the ability to improve lactation performance in poor performing breeds. To enhance the lactation performance of dairy animals, the knowledge of gene expression along with SNP profiling and biological pathways and mechanisms that promotes the mammary gland development and lactation is important.

High-throughput RNA sequencing technologies have provided unprecedented prospects in functional and comparative genomic research, including gene expression, genome annotation and pathway analysis, non-coding RNA discovery, and SNP detection and profiling (Bentley, 2006; Morozova and Marra, 2008). It has been widely used to study SNP in cattle breeds like Polish Holstein-Friesian (Pareek et al., 2016), Brangus, Brahman, Nellore, Angus, and Holstein (Dias et al., 2017) and indigenous cattle breeds like Simmental bull, Xuanhan bull, and Shuxuan bull (Wang et al., 2018).

Kashmiri and Jersey cattle are two important milk animals of the Indian northern state, Kashmir which contributes significantly to the total milk production in the state. Kashmiri indigenous cattle are small, hardy, and well adapted to the hilly areas of this region and differ greatly from Jersey cattle in dairy production traits. Whereas Jersey is a well-established exotic dairy breed which has been utilized in crossbreeding programs to enhance the milk production capability of Kashmiri cattle (Bhat et al., 2019).

In the present study, we used the transcriptome data of Jersey and Kashmiri cattle with the objective to identify the single nucleotide polymorphism in coding regions that were associated with milk production traits. Additionally, the study is aimed to benefit the marker-assisted selection programs by cataloging the differential SNP profile for the improvement of milk production in Kashmiri indigenous cattle.

## Materials and Methods

Transcriptome data of the Jersey and Kashmiri cattle were downloaded from NCBI SRA database with accession number SRR6324372. The samples were obtained at three different lactation stages from three Jersey and three Kashmiri cattle’s breed in similar conditions at Sher-e-Kashmir University of Agricultural Sciences and Technology dairy farm, Mountain Livestock Research Institute (MLRI), Kashmir, India. The sample collection from the healthy animals including sample preparation and RNA sequencing were explained in the study by Bhat et al. (2019). Read quality control was assessed using FASTQC program v0.11.1 (Andrews, 2010). After preprocessing, filtering of low-quality sequences and adaptor trimming were performed using Cutadapt v3.40 (Martin, 2011), high-quality sequencing reads that passed thresholds (PhredScore > 30) were assembled for SNP discovery analysis. A collection of over 40 million high-quality clean reads were obtained for each sample. The cleaned reads were mapped to reference genome assembly ARS-UCD1.2.99 using HISAT2 (Kim et al., 2019). The data preprocessing steps recommended in the GenomeAnalysisToolkit (GATK) best practices workflow was performed before variant identification (Poplin et al., 2018). PCR duplicates were marked with the MarkDuplicates from Picard tools (Picard toolkit, 2019). We also performed local realignment around InDels, checked intron-exon junctions, and recalibrated the base quality scores with GATK. Two different variant callers were used to perform SNP and INDELs discovery across eight Jersey and nine Kashmiri transcriptome samples separately: (i) GATK using the HaplotypeCaller tool in multi-sample calling mode (modality “GATK”); (ii) mpileup from SAMtools v1.4 (Li et al., 2009) in multisample calling mode using default parameters. Final set for analysis contains SNPs and InDelscommon in both datasets.

Bovine genetic variants from dbSNP 2.0 build 153 dated: Aug 8 2019 were incorporated in SNP calling to populate the RS_ID column of the known SNPs. Filtering [base quality score (Q-Score) > 30, mapping quality > 30, and minimum depth > 10] of generated variants and annotation were performed using VCFtools version 0.1.8 and SnpEff program v4.1. To further evaluate the biological significance of the genes with high-impact variations, the KEGG pathway enrichment analysis was performed using nKOBAS server version 3 (Xie et al., 2011; Kanehisa et al., 2021).

## Results

In our previous study after analyzing the inter- and intrastage gene expression profiling between Jersey and Kashmiri cattle, we observed a vast diversity in terms of gene expression while huge similarity between the breeds was also witnessed. Differentially expressed genes in both Kashmiri and Jersey were enriched for multicellular organismal process, receptor activity, catalytic activity, signal transducer activity, macromolecular complex, and developmental processes. Most of the identified pathways responsible for milk production in Jersey include JAK-STAT, p38 MAPK, and PI3 kinase whereas antioxidant genes like RPLPO and RPS28 are highly expressed in Kashmiri cattle (Bhat et al., 2019). The present study based on SNP profiling in coding regions showed interbreed potential difference between Jersey and Kashmiri cattle. The difference in SNPs can help to understand its potential role in controlling the milk production between the two breeds.

### Quality Control, Mapping, and Posttreatment

**FIGURE 1:**
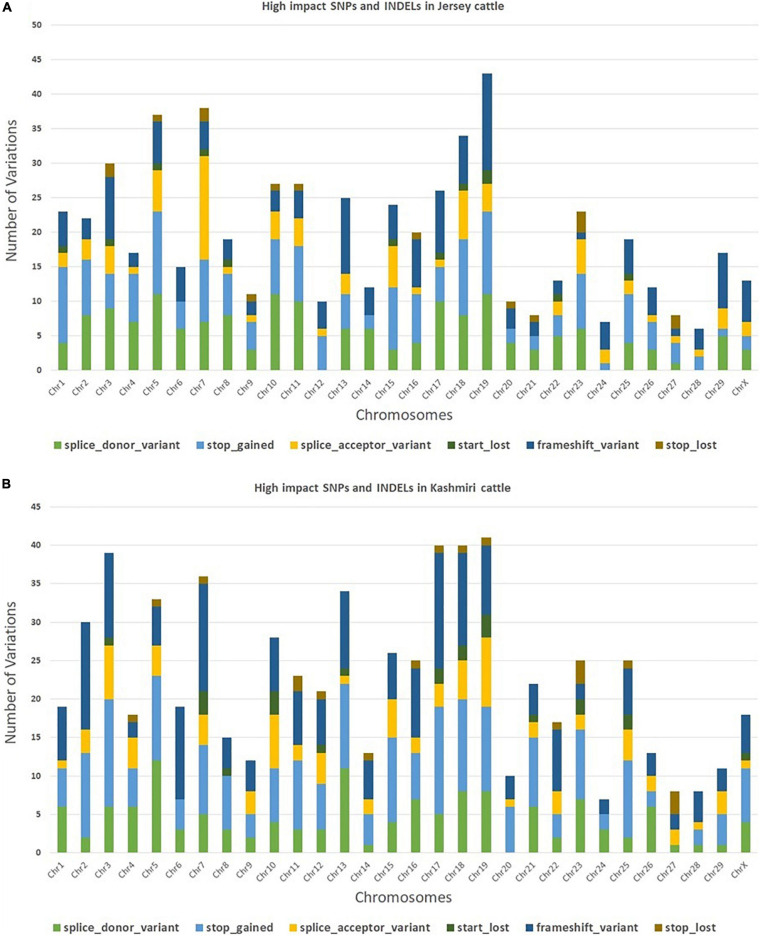
Chromosomal distribution of high impact SNPs and Indels in Jersey **(A)** and Kashmiri **(B)** cattle.

The trimming process removed 0.21% of reads and only 4.2% of duplicated reads were rejected. At the mapping step, around 99.7% of the reads were mapped against the reference genome (Bostaurus assembly ARS-UCD1.2.99) (Bhat et al., 2021). A total of 657,299 and 650,556 SNPs and INDELs were identified from Jersey and Kashmir cattle. Chromosomal distribution of SNPs and INDELs were provided in Supplementary Table 1, and variant types were provided in Supplementary Tables 2, 3. Transitions to transversions ratio (Ts/Tv) in both breeds were around 2.6, with 2,047,112 transitions and 761,864 transversions in Jersey and 1,989,741 transitions and 740,123 transversions in Kashmir cattle. A total of 34.37% missense, 0.296% non-sense, and 65.334% silent mutation were identified in Kashmiri cattle, and 37.701% missense, 0.409% non-sense, and 61.89% silent mutations were identified in Jersey cattle. The common SNPs were filtered out and further analyses were carried out on high-impact SNPs and INDELs specific to Jersey (442 SNPs and 169 INDELs) and Kashmiri (464 SNPs and 220 INDELs) cattle. A total of 351 high-impact variations were identified as frameshift, 400 stop-gained variations, 40 stop lost, 38 start lost, and one SNP in the 5′-UTR region (Supplementary Tables 4, 5). SNP distribution on different chromosomes in both Jersey and Kashmiri cattle are shown in Figure 1.

### Analysis of Genes With SNPs and INDELs

**FIGURE 2:**
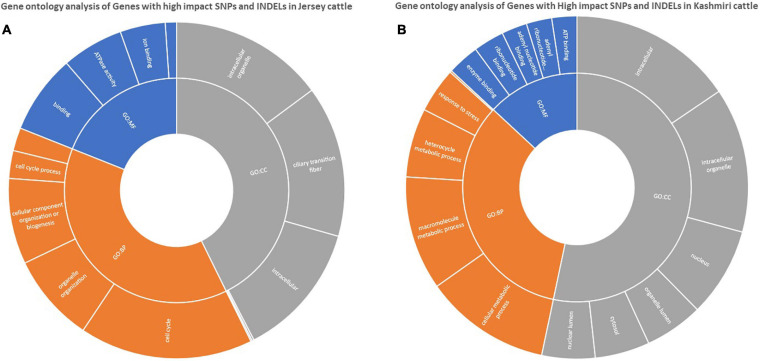
GO enrichment analysis of genes with high impact variations in Jersey **(A)** and Kashmiri **(B)** cattles.

Functional annotation suggests the enrichment (*p*-value < 0.05) of signaling pathways (like AMP activated protein kinase (AMPK) and tumor growth factor (TGF) beta), insulin resistance, and high antigen processing in Kashmiri cows. Jersey cattle enrichment analysis shows genes with high-impact SNPs were involved mainly in nucleic acid (specifically pyrimidine metabolism) and nucleotide (specifically glycine, serine, and threonine) metabolism pathways. Interestingly, the activity of primary immunodeficiency pathway, nucleotide excision repair pathway, glycerolipid metabolism, and phosphatidylinositol signaling pathway were significantly enriched in Jersey cattle (Table 1). Gene ontology (GO) analysis strongly suggests (FDR corrected *p*-value < 0.05) genes with SNPs in Kashmiri cows were mainly involved in the binding process (enzyme and ribonucleotide binding) and antigen processing (Figure 2). In Jersey cattle, mutated genes were involved mainly in ion binding and ATPase activity (Figure 2).

**TABLE 1:** Pathways affected by high-impact SNPs in Jersey **(A)** and Kashmiri **(B)** cattle.

A. Enriched pathways in Jersey cattle.			
Pathway	*p*-value	Input number	Input
Antifolate resistance	0.002919	5	ENSBTAG00000047764, ENSBTAG00000007599, ENSBTAG00000023309, ENSBTAG00000045751, and ENSBTAG00000031500
Nucleotide excision repair	0.005815	5	ENSBTAG00000032527, ENSBTAG00000021773, ENSBTAG00000014595, ENSBTAG00000007362, and ENSBTAG00000014727
ABC transporters	0.008163	5	ENSBTAG00000047764, ENSBTAG00000045751, ENSBTAG00000006921, ENSBTAG00000023309, and ENSBTAG00000002747
Glycerolipid metabolism	0.013722	5	ENSBTAG00000021695, ENSBTAG00000012060, ENSBTAG00000045746, ENSBTAG00000018201, and ENSBTAG00000011917
Glycine, serine, and threonine metabolism	0.022811	4	ENSBTAG00000021695, ENSBTAG00000031814, ENSBTAG00000031500, and ENSBTAG00000004510
Phosphatidylinositol signaling system	0.026138	6	ENSBTAG00000002010, ENSBTAG00000002350, ENSBTAG00000003809, ENSBTAG00000001030, ENSBTAG00000013116, and ENSBTAG00000020715
Pyrimidine metabolism	0.04586	4	ENSBTAG00000021830, ENSBTAG00000005152, ENSBTAG00000008428, and ENSBTAG00000011527
Primary immunodeficiency	0.048001	3	ENSBTAG00000005280, ENSBTAG00000023144, and ENSBTAG00000006452
**B. Enriched pathways in Kashmiri cattle.**			
Antigen processing and presentation	0.0447	9	ENSBTAG00000045795, ENSBTAG00000006270, ENSBTAG00000002069, ENSBTAG00000038128, ENSBTAG00000019386, ENSBTAG00000012208, ENSBTAG00000020116, ENSBTAG00000005182, and ENSBTAG00000019588
Innate immune system	0.002183	46	ENSBTAG00000047856, ENSBTAG00000033662, ENSBTAG00000015520, ENSBTAG00000002902, ENSBTAG00000010639, ENSBTAG00000046188, ENSBTAG00000020772, ENSBTAG00000021474, ENSBTAG00000038797, ENSBTAG00000031641, ENSBTAG00000045861, ENSBTAG00000012467, ENSBTAG00000005660, ENSBTAG00000003401, ENSBTAG00000003774, ENSBTAG00000019020, ENSBTAG00000017866, ENSBTAG00000017401, ENSBTAG00000018403, ENSBTAG00000016415, ENSBTAG00000015187, ENSBTAG00000005574, ENSBTAG00000010951, ENSBTAG00000027684, ENSBTAG00000002854, ENSBTAG00000023144, ENSBTAG00000049346, ENSBTAG00000001014, ENSBTAG00000016021, ENSBTAG00000001609, ENSBTAG00000020433, ENSBTAG00000012780, ENSBTAG00000018661, ENSBTAG00000006270, ENSBTAG00000021144, ENSBTAG00000007964, ENSBTAG00000002069, ENSBTAG00000019386, ENSBTAG00000012208, ENSBTAG00000020116, ENSBTAG00000005182, ENSBTAG00000044040, ENSBTAG00000053934, ENSBTAG00000047699, ENSBTAG00000019250, and ENSBTAG00000003121
TGF beta signaling pathway	0.008891	12	ENSBTAG00000004154, ENSBTAG00000047856, ENSBTAG00000008300, ENSBTAG00000018127, ENSBTAG00000020053, ENSBTAG00000015720, ENSBTAG00000010662, ENSBTAG00000021145, ENSBTAG00000001609, ENSBTAG00000019289, ENSBTAG00000010649, and ENSBTAG00000006919
Insulin resistance	0.011672	8	ENSBTAG00000009175, ENSBTAG00000016336, ENSBTAG00000017024, ENSBTAG00000001400, ENSBTAG00000017866, ENSBTAG00000017639, ENSBTAG00000039958, and ENSBTAG00000002917
Osteoclast differentiation	0.017121	8	ENSBTAG00000007213, ENSBTAG00000007531, ENSBTAG00000001400, ENSBTAG00000019250, ENSBTAG00000021358, ENSBTAG00000003305, ENSBTAG00000021842, and ENSBTAG00000001609
AMPK signaling pathway	0.018729	8	ENSBTAG00000002883, ENSBTAG00000009175, ENSBTAG00000000754, ENSBTAG00000017024, ENSBTAG00000001400, ENSBTAG00000017866, ENSBTAG00000039958, and ENSBTAG00000002917
Endocytosis	0.046451	11	ENSBTAG00000019386, ENSBTAG00000019072, ENSBTAG00000002069, ENSBTAG00000007964, ENSBTAG00000005182, ENSBTAG00000020116, ENSBTAG00000027684, ENSBTAG00000018959, ENSBTAG00000015720, ENSBTAG00000007120, and ENSBTAG00000010543

## Discussion

Kashmiri cattle are poor performing and differ greatly from Jersey in dairy production characteristics. A comprehensive phenotypic analysis with respect to milk production traits was carried out in our previous study (Bhat et al., 2019) which showed higher milk yield and protein content for Jersey cattle as compared with the Kashmiri indigenous cattle. The average milk production of Jersey and Kashmiri cattle varies between 6 and 10 kg/day and 3 and 5 kg/day, respectively. The protein content in Jersey cattle ranged from 2.91 to 3.36%, and the corresponding values for Kashmiri cattle were 2.81–3.21%. The Jersey is among the top milk producer cattle breeds and is routinely used to upgrade the milk-producing capacity of the Kashmiri cattle by crossbreeding practices. It becomes imperative to understand the difference at multiple levels such as gene expression and SNPs in the coding and regulatory region of these breeds. For this purpose, we have analyzed RNA-seq data and performed the comparative study between both the breeds. A SNP detection analysis was performed using sequencing reads from nine Jersey and eight Kashmiri cows to determine putative polymorphisms in genes involved in the milk production. A total 442 and 464 high-impact SNPs were identified from Jersey and Kashmir cattle, respectively. Out of all the highly confident SNPs identified, a significant portion of them (34.37 and 37.701%) were missense mutations distributed in 29 chromosomes. Furthermore, the functional analysis of the genes showed that they were involved in several crucial biological and physiological processes in lactation. We observed TGF-beta, AMPK, and endocytosis show higher activity in Kashmiri cattle compared with Jersey breed. It is reported that TGF-β1 expression increases in MECs during of mammary gland involution in mouse (Atwood et al., 1995), goat (Wareski et al., 2001), sow (Motyl et al., 2001), and cow (Zarzyńska et al., 2007). TGF-β1 plays a key role in the involution of the bovine mammary gland by prompting apoptosis and autophagy in bovine mammary luminal epithelial cells (Kolek et al., 2003; Gajewska et al., 2005). Apoptosis is the foremost kind of cell death responsible for the involution of the bovine mammary gland, but type II programmed cell death (PCD II) is also observed: autophagic cell death. The MEC apoptosis is a marked indication of ending milking at the beginning of the dry period (Zarzyńska and Motyl, 2008). We presume the shorter lactation length in Kashmiri cattle could be one of the reasons for the increased expression of apoptotic signaling pathway toward the beginning of dry period. Moreover, it is known that some milk trait genes (e.g., genes in apoptosis pathway) are not solely expressed in MECs but also by other cell types like leukocytes (Boutinaud et al., 2015).

The levels of TGF-β1 and TGF-β2 decrease with advancing chronological age, and colostrum contains the highest levels for both (Frost et al., 2014). The pronounced role of AMPK is to regulate catabolic and anabolic processes. Negative energy balance (NEB) is generally witnessed in milking cows particularly during lactation. Increase in ADP or AMP is usually related with a NEB. It has been found that during maximum lactation, the energy intake in dairy cows is not able to fulfill the requirement for milk production. The AMPK signaling has been found greatly active throughout this period, which results in significant reduction in the milk of cows (Eastham et al., 1988). AMPK regulates lipid metabolism by altering the transcription of the lipogenic gene and the posttranslational modification of the major enzymes involved in lipid synthesis. In addition, AMPK activation was found to repress global protein synthesis *via* inhibition of mTOR signals in bovine mammary epithelial cells (Appuhamy et al., 2014). These changes along with the effect of lower plane of nutrition during formative phase of the animals and consequent poor body condition may contribute to the lower concentrations of total milk proteins in Kashmiri cattle. This statement however, needs to be validated. Similar studies were reported in lactating goats (Cai et al., 2020).

An endocytosis pathway was found to be enriched in the milk of Kashmiri cattle. The mechanism for internalization (endocytosis) and release (exocytosis) are governed by the physiological phase of the mammary gland (McShane and Zerial, 2008). Furthermore, Truchet and Ollivier-Bousquet (2009) reported that the mammary epithelium enrichment organizes the absorption of milk precursors and transport of milk components to produce milk of reasonably constant composition, provided the mammary gland is healthy.

Several important metabolic pathways that were found to be enriched exclusively in Jersey cattle include glycolipid, pyrimidine, and amino acid metabolism (specifically glycine, serine, and threonine) and other important pathways like phosphatidylinositol signaling, Antifolate resistance, ABC transporters pathway, and nucleotide excision repair. Amino acid (AA) metabolism plays a critical role in milk production by regulating maternal endocrine status, blood flow through the lactating mammary gland, and activation of mechanistic (mammalian) target rapamycin (mTOR) signaling (Wu et al., 2010). The higher activity of glycine, serine, and threonine metabolism pathways in Jersey cattle may be particularly important in lactation initiation and higher milk production (Li and Jiang, 2019). TCA cycle and glycine, serine, and threonine metabolism pathways are the most important and work together in the mammary gland for lactation initiation (Sun et al., 2015, 2017). It is a known fact that AAs significantly contribute to liver gluconeogenesis in early lactation. Glycine, serine, and alanine, comprised more than 65% of the liver uptake of glucogenic AA during the periparturient period (Larsen and Kristensen, 2009, 2012). In Jersey cattle, the phosphatidylinositol 3-kinase (PI3K)/protein kinase B (Akt) signaling pathway emerged as the most enriched pathway. PI3K-Akt pathway mediates glucose absorption into cells and is important for normal insulin-mediated glucose metabolism (Taniguchi et al., 2006). Since Jersey cattle produce milk in higher quantities than Kashmiri cattle, it requires a high-energy source. The enrichment of glycerolipid metabolic pathway in Jersey *via* glycerolipid pathway converts glycerol into glucose thus providing sufficient energy for maintaining high milk production (Sun et al., 2017).

In Kashmiri cattle, we found variations in the major histocompatibility complex (MHC) region. In cattle, polymorphism in the MHC class II genes influences both the magnitude and specificity of antigen-specific T cells to various diseases like mastitis (Hameed et al., 2006). In our previous study, we found that a wide range of proteins like apelin, acid glycoprotein, CD14 antigens, and lactoferrin, involved in immune response and host defense were highly expressed in mammary gland of Kashmiri cattle (Bhat et al., 2020). The role of the MHC in immune response makes a MHC an attractive candidate gene to study associations with disease resistance or susceptibility in cattle. High variability in many MHC genes plays a role in the recognition of bacteria (Apanius et al., 1997). Hedrick and Kim (1999) demonstrate the relation between MHC variation and resistance to bacterial infections. There are several well-documented cases in which specific MHC haplotypes or genotypes provide resistance to bacteria (Hill et al., 1991; Carrington et al., 1999).

We also found variations in genes regulating TGF-β (SMAD4, BMP7) and mTOR signaling in Kashmiri cattle (Supplementary Figures 1, 2) (Bhat et al., 2017). SMAD4 proteins serve as crucial components of TGF-β signaling, which negatively regulates cell growth and promotes apoptosis of epithelial cells. The rate of decline in milk yield with stage of lactation is strongly influenced by the rate of cell death by apoptosis in the lactating gland (Stefanon et al., 2002). Such changes depict the early backdrop for dryness in Kashmiri cattle. SMAD4 has been found to play important roles in various biological processes. In humans, genetic variants in the SMAD4 have been found to play a protective role in various types of cancers (Wu et al., 2010). A high-impact stop-gained variation on SMAD4 (c.1165C) and frameshift variation on BMP7 (c.693delC) could possibly dysregulate TGF-β pathway in Kashmiri cattle. Further study is required to study the effect of these variations on the TGF-β signaling pathway.

It is widely accepted that mTOR is a key regulator of milk protein synthesis, and most reports were concerned with the role of AAs in the regulation of P-mTOR on Ser2448 in milk protein synthesis (Appuhamy et al., 2012; Apelo et al., 2014). mTORC1 signaling mediates the cell surface level of Wnt receptor frizzled FZD and plays an essential role in embryogenesis and homeostasis Chen et al. (2020). In cattle, the milk protein synthesis was upregulated through activation of the mTOR pathway (Gao et al., 2015). We found double frameshift variations on FZD9 (c.484delG and c.1563delT) in Kashmiri cattle and could be the probable reason for its low milk production.

## Conclusion

The SNP analysis based on RNA sequencing demonstrated a clear distinction between Jersey and Kashmiri cattle in milk production traits. In Kashmiri cattle, the high-impact SNP variants were involved in adaptive immunity and resistance against mammary gland infectious diseases. Whereas, in Jersey cattle, enriched pathways were mainly involved in maintenance of lactation and production. These findings provide insights in genetic variation of the breeds that can be used for genomic selection of the animals.

## Data Availability Statement

Publicly available datasets were analyzed in this study. This data can be found here: https://www.ncbi.nlm.nih.gov/sra/SRR6324372.

## Author Contributions

SA designed the study and wrote the manuscript. BB and MY performed the data analysis. SB and SM helped in the writing of the manuscript. MR and MI helped in data interpretation and analysis. RS and NG did proofreading of the manuscript. All authors revised and approved the final version of the manuscript.

## Conflict of Interest

The authors declare that the research was conducted in the absence of any commercial or financial relationships that could be construed as a potential conflict of interest.

## Publisher’s Note

All claims expressed in this article are solely those of the authors and do not necessarily represent those of their affiliated organizations, or those of the publisher, the editors and the reviewers. Any product that may be evaluated in this article, or claim that may be made by its manufacturer, is not guaranteed or endorsed by the publisher.

## References

[B1] Andrews S. (2010). *FastQC**: A Quality Control Tool for High Throughput Sequence Data.* Available online at: http://www.bioinformatics.babraham.ac.uk/projects/fastqc (accessed January 8, 2019).

[B2] Apanius V. Penn D. Slev P. R. Ruff L. R. Potts W. K. (1997). The nature of selection on the major histocompatibility complex. *Crit. Rev. Immunol.* 17 179–224. 9094452 10.1615/critrevimmunol.v17.i2.40

[B3] Apelo S. A. Knapp J. Hanigan M. (2014). Invited review: current representation and future trends of predicting amino acid utilization in the lactating dairy cow. *J. Dairy Sci.* 97 4000–4017. 10.3168/jds.2013-7392 24767883

[B4] Appuhamy J. R. N. Knoebel N. A. Nayananjalie W. D. Escobar J. Hanigan M. D. (2012). Isoleucine and leucine independently regulate mtor signaling and protein synthesis in mac-t cells and bovine mammary tissue slices. *J. Nutr.* 142 484–491. 10.3945/jn.111.152595 22298573

[B5] Appuhamy J. Nayananjalie W. England E. Gerrard D. Akers R. Hanigan M. (2014). Effects of amp-activated protein kinase (ampk) signaling and essential amino acids on mammalian target of rapamycin (mtor) signaling and protein synthesis rates in mammary cells. *J. Dairy Sci.* 97 419–429. 10.3168/jds.2013-7189 24183687

[B6] Arora R. Sharma A. Sharma U. Girdhar Y. Kaur M. Kapoor P. (2019). Buffalo milk 205 transcriptome: a comparative analysis of early, mid and late lactation. *Sci. Rep.* 9:5993. 10.1038/s41598-019-42513-2 PMC6461664 30979954

[B7] Atwood C. S. Ikeda M. Vonderhaar B. K. (1995). Involution of mouse mammary glands in whole organ culture: a model for studying programmed cell death. *Biochem. Biophys. Res. Commun.* 207 860–867. 10.1006/bbrc.1995.1265 7864882

[B8] Auldist M. J. O’brien G. Cole D. Macmillan K. L. Grainger C. (2007). Effects of varying lactation length on milk production capacity of cows in pasture-based dairying systems. *J. Dairy Sci.* 90 3234–3241. 10.3168/jds.2006-683 17582106

[B9] Bentley D. R. (2006). Whole-genome re-sequencing. *Curr. Opin. Genet. Dev.* 16 545–552. 17055251 10.1016/j.gde.2006.10.009

[B10] Bhat B. Ganai N. A. Andrabi S. M. Shah R. A. Singh A. (2017). Tm-aligner: multiple sequence alignment tool for transmembrane proteins with reduced time and improved accuracy. *Sci. Rep.* 7:12543. 10.1038/s41598-017-13083-y PMC5624947 28970546

[B11] Bhat B. Yaseen M. Singh A. Ahmad S. M. Ganai N. A. (2021). Identification of potential key genes and pathways associated with the pashmina fiber initiation using rna-seq and integrated bioinformatics analysis. *Sci. Rep.* 11:1766. 10.1038/s41598-021-81471-6 PMC7815713 33469142

[B12] Bhat S. A. Ahmad S. M. Ibeagha-Awemu E. M. Bhat B. A. Dar M. A. Mumtaz P. T. (2019). Comparative transcriptome analysis of mammary epithelial cells at different stages of lactation reveals wide differences in gene expression and pathways regulating milk synthesis between Jersey and Kashmiri cattle. *PLoS One* 14:e0211773. 10.1371/journal.pone.0211773 PMC6363229 30721247

[B13] Bhat S. A. Ahmad S. M. Ibeagha-Awemu E. M. Mobashir M. Dar M. A. Mumtaz P. T. (2020). Comparative milk proteome analysis of Kashmiri and Jersey cattle identifies differential expression of key proteins involved in immune system regulation and milk quality. *BMC Genomics* 21:161. 10.1186/s12864-020-6574-4 PMC7023774 32059637

[B14] Boutinaud M. Herve L. Lollivier V. (2015). Mammary epithelial cells isolated from milk are a valuable, non-invasive source of mammary transcripts. *Front. Genet.* 6:323. 10.3389/fgene.2015.00323 PMC4623414 26579195

[B15] Cai J. Wang D. Zhao F. Q. Liang S. Liu J. (2020). AMPK-mTOR pathway is involved in glucose-modulated amino acid sensing and utilization in the mammary glands of lactating goats. *J. Anim. Sci. Biotechnol.* 11 1–12. 32166025 10.1186/s40104-020-0434-6 PMC7060552

[B16] Carrington M. Nelson G. W. Martin M. P. Kissner T. Vlahov D. Goedert J. J. (1999). Hlaand hiv-1: heterozygote advantage and b^∗^ 35-cw^∗^ 04 disadvantage. *Science* 283 1748–1752. 10.1126/science.283.5408.1748 10073943

[B17] Chen M. Amado N. Tan J. Reis A. Ge M. Abreu J. G. (2020). Tmem79/mattrin defines a pathway for frizzled regulation and is required for xenopus embryogenesis. *Elife* 9:e56793. 10.7554/eLife.56793 PMC7521923 32924931

[B18] Dekkers J. C. (2004). Commercial application of marker-and gene-assisted selection in livestock: strategies and lessons. *J. Anim. Sci.* 82 E313–E328. 15471812 10.2527/2004.8213_supplE313x

[B19] Dias M. M. Cánovas A. Mantilla-Rojas C. Riley D. G. Luna-Nevarez P. Coleman S. J. (2017). SNP detection using RNA-sequences of candidate genes associated with puberty in cattle. *Genet. Mol. Res*. 16:gmr16019522. 10.4238/gmr16019522 28340271

[B20] Eastham P. R. Smith W. C. Whittemore C. T. Phillips P. (1988). Responses of lactating sows to food level. *Anim. Sci.* 46 71–77. 10.1017/s0003356100003123

[B21] Frost B. L. Jilling T. Lapin B. Maheshwari A. Caplan M. S. (2014). Maternal breast milk transforming growth factor-beta and feeding intolerance in preterm infants. *Pediatr. Res.* 76 386–393. 10.1038/pr.2014.96 24995914 PMC4467901

[B22] Gajewska M. Gajkowska B. Motyl T. (2005). Apoptosis and autophagy induced by tgf-ß1 in bovine 247 mammary epithelial bme-uv1 cells. *J. Physiol. Pharmacol.* 56 143–157. 16077200

[B23] Gao H.-n Hu H. Zheng N. Wang J.-q (2015). Leucine and histidine independently regulate milk protein synthesis in bovine mammary epithelial cells via mtor signaling pathway. *J. Zhejiang Univ. Sci. B* 16 560–572. 10.1631/jzus.b1400337 26055918 PMC4471608

[B24] Georges M. Nielsen D. Mackinnon M. Mishra A. Okimoto R. Pasquino A. T. (1995). Mapping quantitative trait loci controlling milk production in dairy cattle by exploiting progeny testing. *Genetics* 139 907–920. 10.1093/genetics/139.2.907 7713441 PMC1206390

[B25] Goldman A. S. (2000). Modulation of the gastrointestinal tract of infants by human milk. Interfaces and interactions. An evolutionary perspective. *J. Nutr.* 130 426S–431S. 10721920 10.1093/jn/130.2.426S

[B26] Hameed K. G. A. Sender G. Mayntz M. (2006). Major histocompatibility complex polymorphism and mastitis resistance–a review. *Anim. Sci. Pap. Rep.* 24 11–25.

[B27] Hayes B. Goddard M. (2010). Genome-wide association and genomic selection in animal breeding. *Genome* 53 876–883. 10.1139/g10-076 21076503

[B28] Hedrick P. W. Kim T. J. (1999). “Genetics of complex polymorphisms: parasites and maintenance of mhc variation,” in *Evolutionary Genetics From Molecules to Morphology*, eds Singh R. S. Krimbas C. K. (Cambridge: Cambridge University Press).

[B29] Hill A. V. Allsopp C. E. Kwiatkowski D. Anstey N. M. Twumasi P. Rowe P. A. (1991). Common west africanhla antigens are associated with protection from severe malaria. *Nature* 352 595–600. 10.1038/352595a0 1865923

[B58] Kanehisa M. Furumichi M. Sato Y. Ishiguro-Watanabe M. Tanabe M. (2021). KEGG: integrating viruses and cellular organisms. *Nucleic Acids Res.* 49 D545–D551. 10.1093/nar/gkaa970 33125081 PMC7779016

[B30] Kim D. Paggi J. M. Park C. Bennett C. Salzberg S. L. (2019). Graph-based genome alignment and genotyping with HISAT2 and HISAT-genotype. *Nat. Biotechnol.* 37 907–915. 10.1038/s41587-019-0201-4 31375807 PMC7605509

[B31] Kolek O. Gajkowska B. Godlewski M. Motyl T. (2003). Antiproliferative and apoptotic effect of tgf-β1 in bovine mammary epithelial bme-uv1 cells. *Comp. Biochem. Physiol. Part C Toxicol. Pharmacol.* 134 417–430. 10.1016/s1532-0456(02)00249-1 12727291

[B32] Larsen M. Kristensen N. B. (2009). Effect of abomasal glucose infusion on splanchnic amino acid metabolism in periparturient dairy cows. *J. Dairy Sci.* 92 3306–3318. 10.3168/jds.2008-1889 19528608

[B33] Larsen M. Kristensen N. B. (2012). Effects of glucogenic and ketogenic feeding strategies on splanchnic glucose and amino acid metabolism in postpartum transition Holstein cows. *J. Dairy Sci.* 95 5946–5960. 10.3168/jds.2012-5458 22921630

[B34] Li Z. Jiang M. (2019). Metabolomic profiles in yak mammary gland tissue during the lactation cycle. *PLoS One* 14:e0219220. 10.1371/journal.pone.0219220 PMC6611666 31276563

[B35] Li H. Handsaker B. Wysoker A. Fennell T. Ruan J. Homer N. (2009). 1000 genome project data processing subgroup, the sequence alignment/map format and SAMtools. *Bioinformatics* 25 2078–2079. 10.1093/bioinformatics/btp352 19505943 PMC2723002

[B36] Martin M. (2011). Cutadapt removes adapter sequences from highthroughput sequencing reads. *EMBnet J.* 17 10–12. 10.14806/ej.17.1.200

[B37] McShane M. P. Zerial M. (2008). Survival of the weakest: signaling aided by endosomes. *J. Cell Biol.* 182 823–825. 10.1083/jcb.200807165 18779366 PMC2528579

[B38] Mech A. Dhali A. Prakash B. Rajkhowa C. (2008). Variation in milk yield and milk composition during the entire lactation period in Mithun cows (*Bos frontalis*). *Livest. Res. Rural Dev.* 20 56–57.

[B39] Morozova O. Marra M. A. (2008). Applications of next-generation sequencing technologies in functional genomics. *Genomics* 92 255–264. 10.1016/j.ygeno.2008.07.001 18703132

[B40] Motyl T. Gajkowska B. Wojewódzka U. Warȩski P. Rekiel A. Płoszaj T. (2001). Expression of apoptosis-related proteins in involuting mammary gland of sow. *Comp. Biochem. Physiol. Part B Biochem. Mol. Biol.* 128 635–646. 10.1016/s1096-4959(00)00334-1 11290445

[B41] Pareek C. S. Błaszczyk P. Dziuba P. Czarnik U. Fraser L. Sobiech P. (2017). Single nucleotide polymorphism discovery in bovine liver using rna-seq technology. *PLoS One* 12:e0172687. 10.1371/journal.pone.0172687 PMC5325534 28234981

[B42] Pareek C. S. Smoczyński R. Kadarmideen H. N. Dziuba P. Błaszczyk P. Sikora M. (2016). Single nucleotide polymorphism discovery in bovine pituitary gland using RNA-Seq technology. *PLoS One* 11:e0161370. 10.1371/journal.pone.0161370 PMC5015895 27606429

[B43] Picard toolkit (2019). *Broad Institute, GitHub Repository*. Broad Institute. Available online at: http://broadinstitute.github.io/picard/

[B44] Poplin R. Ruano-Rubio V. DePristo M. A. Fennell T. Carneiro M. O. Van der Auwera G. A. (2018). Scaling accurate genetic variant discovery to tens of thousands of samples. *BioRxiv* [Preprint]. 10.1101/201178 BioRxiv: 201178.

[B45] Reinhardt T. A. Lippolis J. D. (2006). Bovine milk fat globule membrane proteome. *J. Dairy Res.* 73 406–416. 10.1017/s0022029906001889 16834814

[B46] Séverin S. Wenshui X. (2005). Milk biologically active components as nutraceuticals. *Crit. Rev. Food Sci. Nutr.* 45 645–656. 10.1080/10408690490911756 16371332

[B47] Stefanon B. Colitti M. Gabai G. Knight C. H. Wilde C. J. (2002). Mammary apoptosis and lactation persistence in dairy animals. *J. Dairy Res.* 69 37–52. 10.1017/s0022029901005246 12047109

[B48] Sun H. Z. Shi K. Wu X. H. Xue M. Y. Wei Z. H. Liu J. X. (2017). Lactation-related metabolic mechanisms investigated based on mammary gland metabolomics and 4 biofluids’ metabolomics relationships in dairy cows. *BMC Genomics* 18:936. 10.1186/s12864-017-4314-1 PMC5712200 29197344

[B49] Sun H. Z. Wang D. M. Wang B. Wang J. K. Liu H. Y. Guan L. L. (2015). Metabolomics of four biofluids from dairy cows: potential biomarkers for milk production and quality. *J. Proteome Res.* 14 1287–1298. 10.1021/pr501305g 25599412

[B50] Taniguchi C. M. Emanuelli B. Kahn C. R. (2006). Critical nodes in signalling pathways: insights into insulin action. *Nat. Rev. Mol. Cell Biol.* 7 85–96. 10.1038/nrm1837 16493415

[B51] Truchet S. Ollivier-Bousquet M. (2009). Mammary gland secretion: hormonal coordination of endocytosis and exocytosis. *Animal* 3 1733–1742. 10.1017/s1751731109990589 22443558

[B52] Wang W. Wang H. Tang H. Gan J. Shi C. Lu Q. (2018). Genetic structure of six cattle populations revealed by transcriptome-wide SNPs and gene expression. *Genes Genom.* 40 715–724. 10.1007/s13258-018-0677-1 PMC6015124 29934811

[B53] Wareski P. Motyl T. Ryniewicz Z. Orzechowski A. Gajkowska B. Wojewodzka U. (2001). Expression of apoptosis-related proteins in the mammary gland of goats. *Small Rumin. Res.* 40 279–289. 10.1016/s0921-4488(01)00178-x 11323213

[B54] Wu D. M. Zhu H.-X. Zhao Q.-H. Zhang Z.-Z. Wang S.-Z. Wang M.-L. (2010). Genetic variations in the smad4 gene and gastric cancer susceptibility. *World J. Gastroenterol.* 16:5635. 10.3748/wjg.v16.i44.5635 PMC2992684 21105199

[B55] Xie C. Mao X. Huang J. Ding Y. Wu J. Dong S. (2011). KOBAS 2.0: a web server for annotation and identification of enriched pathways and diseases. *Nucleic Acids Res.* 39 W316–W322. 21715386 10.1093/nar/gkr483 PMC3125809

[B56] Zarzyńska J. Motyl T. (2008). Apoptosis and autophagy in involuting bovine mammary gland. *J. Physiol. Pharmacol.* 59(Suppl. 9) 275–288. 19261986

[B57] Zarzyńska J. Gajkowska B. Wojewódzka U. Dymnicki E. Motyl T. (2007). Apoptosis and autophagy in involuting bovine mammary gland is accompanied by up-regulation of TGF-beta1 and suppression of somatotropic pathway. *Polish J. Vet. Sci.* 10 1–9. 17388018

