# Supplementary figures and images for: SNPs in Mammary Gland Epithelial Cells Unraveling Potential Difference in Milk Production Between Jersey and Kashmiri Cattle Using RNA Sequencing
Source: Front Genet. 2021 Aug 3;12:666015. doi: 10.3389/fgene.2021.666015 (PMC8369411; doi:10.3389/fgene.2021.666015)

**Figure 1. Genes with high impact (in RED) in TGF-Beta Signaling pathway.**

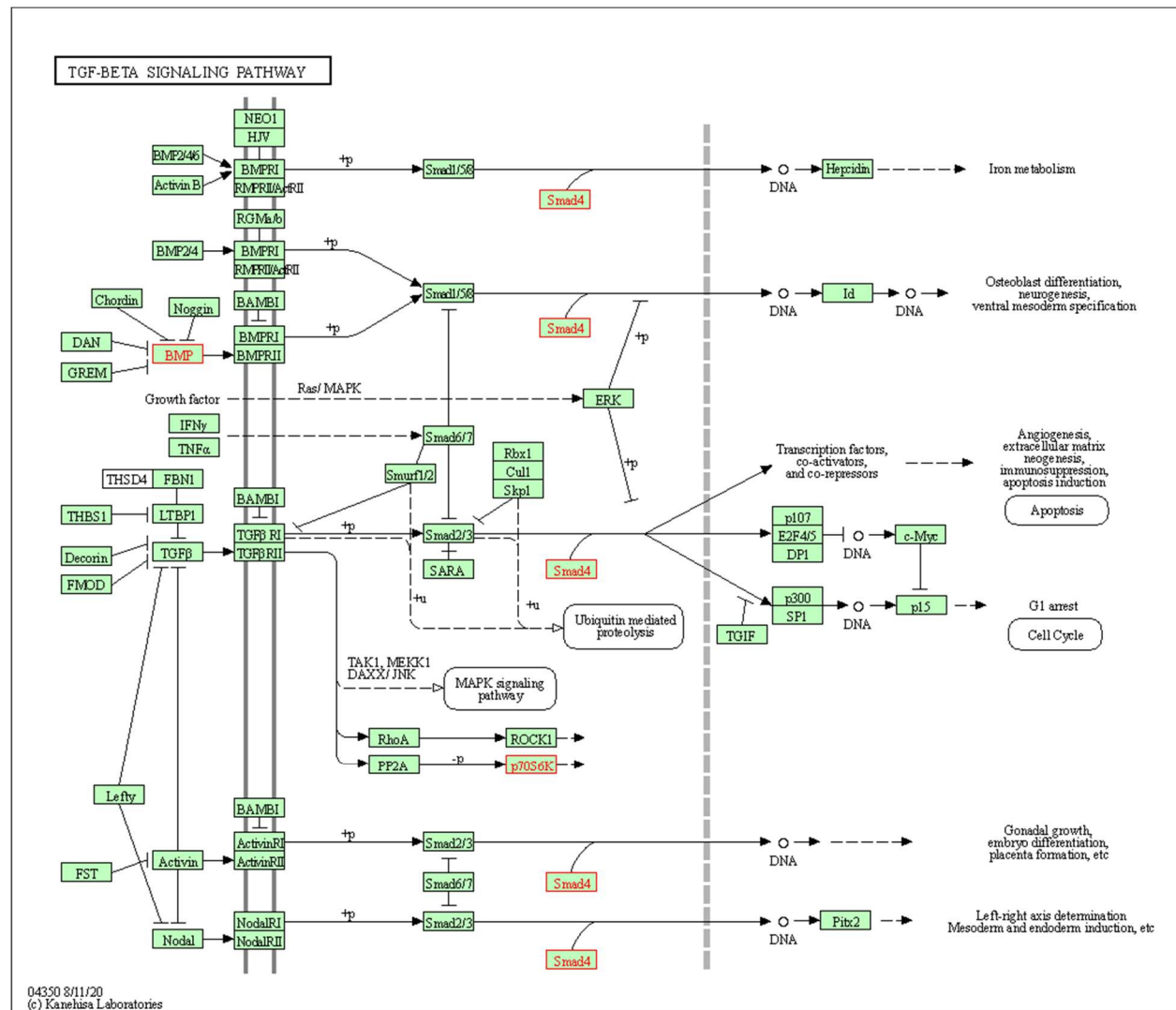

Supplement: Supplementary file 1 [file Image_1.pdf]

Figure 2. Genes with high impact (in RED) in mTOR Signaling pathway.

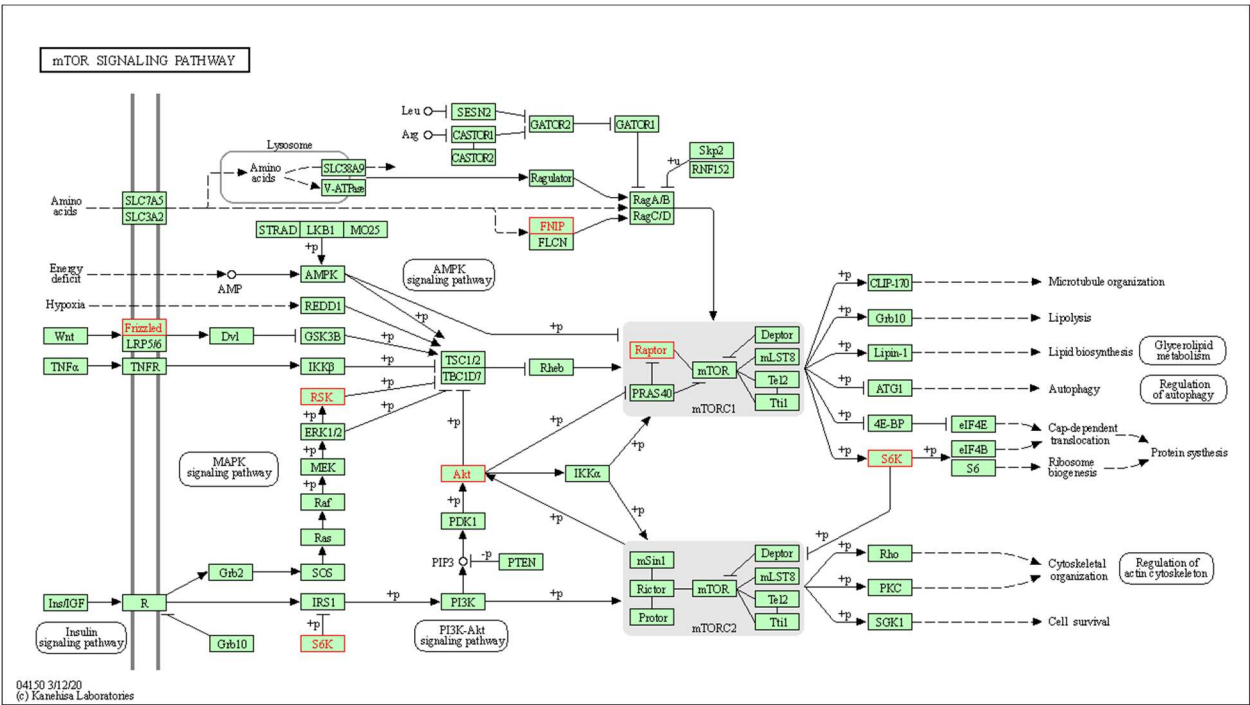

Supplement: Supplementary file 2 [file Image_2.pdf]
