# Supplementary material for: SNPs in Mammary Gland Epithelial Cells Unraveling Potential Difference in Milk Production Between Jersey and Kashmiri Cattle Using RNA Sequencing
Source: Front Genet. 2021 Aug 3;12:666015. doi: 10.3389/fgene.2021.666015 (PMC8369411; doi:10.3389/fgene.2021.666015)
Supplement: Supplementary file 3 [file Table_1.pdf]

Table 1. Variant rate details

Chromosome	Length	Variants in Kashmiri cattle	Variants in Jersey cattle
1	158,534,110	30,097	35,317
2	136,231,102	26,434	24,997
3	121,005,158	31,116	33,952
4	120,000,601	23,054	23,871
5	120,089,316	35,336	34,366
6	117,806,340	22,314	24,024
7	110,682,743	24,548	32,396
8	113,319,770	20,352	22,172
9	105,454,467	18,604	17,295
10	103,308,737	26,653	23,783
11	106,982,474	31,692	34,016
12	87,216,183	12,603	10,941
13	83,472,345	26,848	30,556
14	82,403,003	16,661	15,989
15	85,007,780	21,565	21,535
16	81,013,979	23,381	24,587
17	73,167,244	21,657	15,613
18	65,820,629	31,903	29,104
19	63,449,741	34,103	32,665
20	71,974,595	12,720	10,797
21	69,862,954	17,663	21,050
22	60,773,035	19,178	19,899
23	52,498,615	24,244	19,636
24	62,317,253	11,105	8,881
25	42,350,435	24,471	22,321
26	51,992,305	13,960	10,982
27	45,612,108	8,316	8,463
28	45,940,150	9,126	16,607
29	51,098,607	11,702	11,707
**Total Variants**		**631,406**	**637,522**
