# Supplementary material for: SNPs in Mammary Gland Epithelial Cells Unraveling Potential Difference in Milk Production Between Jersey and Kashmiri Cattle Using RNA Sequencing
Source: Front Genet. 2021 Aug 3;12:666015. doi: 10.3389/fgene.2021.666015 (PMC8369411; doi:10.3389/fgene.2021.666015)
Supplement: Supplementary file 4 [file Table_2.pdf]

Table 2. SNP type in Kashmir cattle

Type	Percent
HIGH	0.105%
LOW	6.84%
MODERATE	3.419%
MODIFIER	89.637%

Type	Percent
MISSENSE	34.37%
NONSENSE	0.296%
SILENT	65.334%
