# Supplementary material for: SNPs in Mammary Gland Epithelial Cells Unraveling Potential Difference in Milk Production Between Jersey and Kashmiri Cattle Using RNA Sequencing
Source: Front Genet. 2021 Aug 3;12:666015. doi: 10.3389/fgene.2021.666015 (PMC8369411; doi:10.3389/fgene.2021.666015)
Supplement: Supplementary file 5 [file Table_3.pdf]

**Table 3. SNP type in Jersey cattle**

| Type | Percent |
|---|---|
| HIGH | 0.108% |
| LOW | 4.544% |
| MODERATE | 2.594% |
| MODIFIER | 92.754% |

| Type | Percent |
|---|---|
| MISSENSE | 37.701% |
| NONSENSE | 0.409% |
| SILENT | 61.89% |
